# Child health services and armed conflict in Tigray, North Ethiopia: a community-based study

**DOI:** 10.1186/s13031-023-00545-6

**Published:** 2023-10-05

**Authors:** Mache Tsadik, Hailay Gebretnsae, Asefa Ayalew, Akeza Awealom Asgedom, Aregawi Gebreyesus, Tigist Hagos, Marta Abrha, Kiros Weldegerima, Birikti Abrha, Gelawdiwos Gebre, Mulubrhan Hagos, Rie Esayas, Mezgebe Gebregeorgis, Hailay Abrha Gesesew, Afework Mulugeta

**Affiliations:** 1 School of Public Health, College of Health Sciences, Mekelle University, Tigray, 231 Ethiopia; 2 Tigray Health Research Institute, Tigray, 07 Ethiopia; 3 School of Medicine, College of Health Sciences, Mekelle University, Tigray, 231 Ethiopia; 4 School of Midwifery, College of Health Sciences, Mekelle University, Tigray, 231 Ethiopia; 5 Tigray Regional Health Bureau, Tigray, 07 Ethiopia; 6 Institute of population studies, Mekelle University, Tigray, 231 Ethiopia; 7 Research Centre for Public Health, Equity and Human Flourishing, Torrens University Australia, Adelaide, SA 5000 Australia

**Keywords:** Child health, Armed-conflict, Vaccines, Immunization, Childhood illness, Tigray

## Abstract

**Background:**

Access to basic health services, notably child health services, is severely hampered by the armed conflict in Tigray, North Ethiopia. Little is known regarding the impacts of the armed conflict during the war in Tigray on access to child health services. The current study investigates the impact of the armed conflict on the utilization of child health services in Tigray.

**Methods:**

4,381 caregivers from randomly recruited households (HHs) with at least one child younger than 1 year old participated in a community-based cross-sectional survey. We collected data on childhood immunizations and illness-related treatment seeking from August 4 to 20, 2021. We describe data using frequency and percentage and carry out an internal comparison among the study participants using chi-square tests.

**Results:**

4,381 children under the age of one included in the study. In total, 39% of infants received no basic vaccines, 61.3% of the children under the age of one received at least one vaccine, and 20% received all the vaccinations recommended for their age. About 61% of children were affected by at least one childhood ailments where majority of them were from rural areas. Mothers who did not seek postnatal care (PNC) were responsible for more than 75% of reported childhood illnesses.

**Conclusions:**

A sizable portion of children were unvaccinated and had at least one childhood sickness while the war was in progress. Particularly, people who live in rural areas reported a higher percentage of children’s illnesses but a lower use of child health services. To lower childhood morbidity and mortality in the besieged area, such as Tigray, local to global actors need to get coordinated and warrying parties should stop weaponization of vaccination healthcare services.

**Supplementary Information:**

The online version contains supplementary material available at 10.1186/s13031-023-00545-6.

## Introduction

Armed conflicts are frequently characterized by damage to health infrastructure and forced migration, injury, or death of health workers which interrupt the medical supply chains and delivery of health services [1].Communities affected by war are excluded from the social and economic advancement of the world, often struggling to survive. Children are among the most vulnerable population groups during conflict [2].

It is evident that vaccinations administered during the first year of life significantly reduce childhood morbidity and mortality [3]. However, one in six children who live in violent conflicts have less access to healthcare, and outbreaks of diseases that can be prevented by vaccination are frequently recorded in conflict-affected areas [4, 5]. Children who reside in such environments are vulnerable to the vaccine preventable illnesses, malaria, acute starvation, violence, and psychosocial harm [6]. About 30% of deaths among children under the age of five were brought on by the estimated 19.9 million infants who were not given routine immunizations [2].

The Tigray health system, once hailed as one of the best in Ethiopia, was on the verge of collapse due to the war that began on 4 November 2020. Led by the Ethiopian National Defence Forces, allied forces including Amhara special forces and Amhara militia, and Foreign Eritrean army attacked Tigray from multiple fronts; and Tigray Defence Forces (TDF) were the defending forces on behalf of the Tigray Regional government and people [7]. Yet to be investigated how disastrous the war was, the war atrociously impacted the entire population, and children are the most vulnerable war victims. Children are frequently undernourished and at danger of dying from diseases that can be prevented or treated by vaccination in settings when basic services are disrupted and livelihoods are destroyed [8]. The morbidity and death of children in Tigray are anticipated to significantly rise in conjunction with the imposed blockade and war, which has resulted in a shortage of food and medical supplies, resulting in an unavoidable generation gap in Tigray’s demography. In the current study, the impact of childhood illnesses, the need for treatment seeking, and the use of child health services during the Tigray War are evaluated. Governmental, non-governmental groups and other actors will use the study’s findings as a foundation when rebuilding child health services in particular and the overall health care system in general.

## Methods

### Study area and setting

Tigray is one of the ten regional states in Ethiopia, with a projected population of 7.3 million in 2022 [9]. While all healthcare institutions at all levels were essentially functional before the war broke out, more than 70% of the health facilities were intentionally damaged following the conflict [10]. The study included participants from both urban and rural community settings. Urban areas are places with 2,000 or more residents, as defined by the Ethiopian Statistical Agency. Rural areas are any areas that are not categorized as urban.

**Fig. 1:**
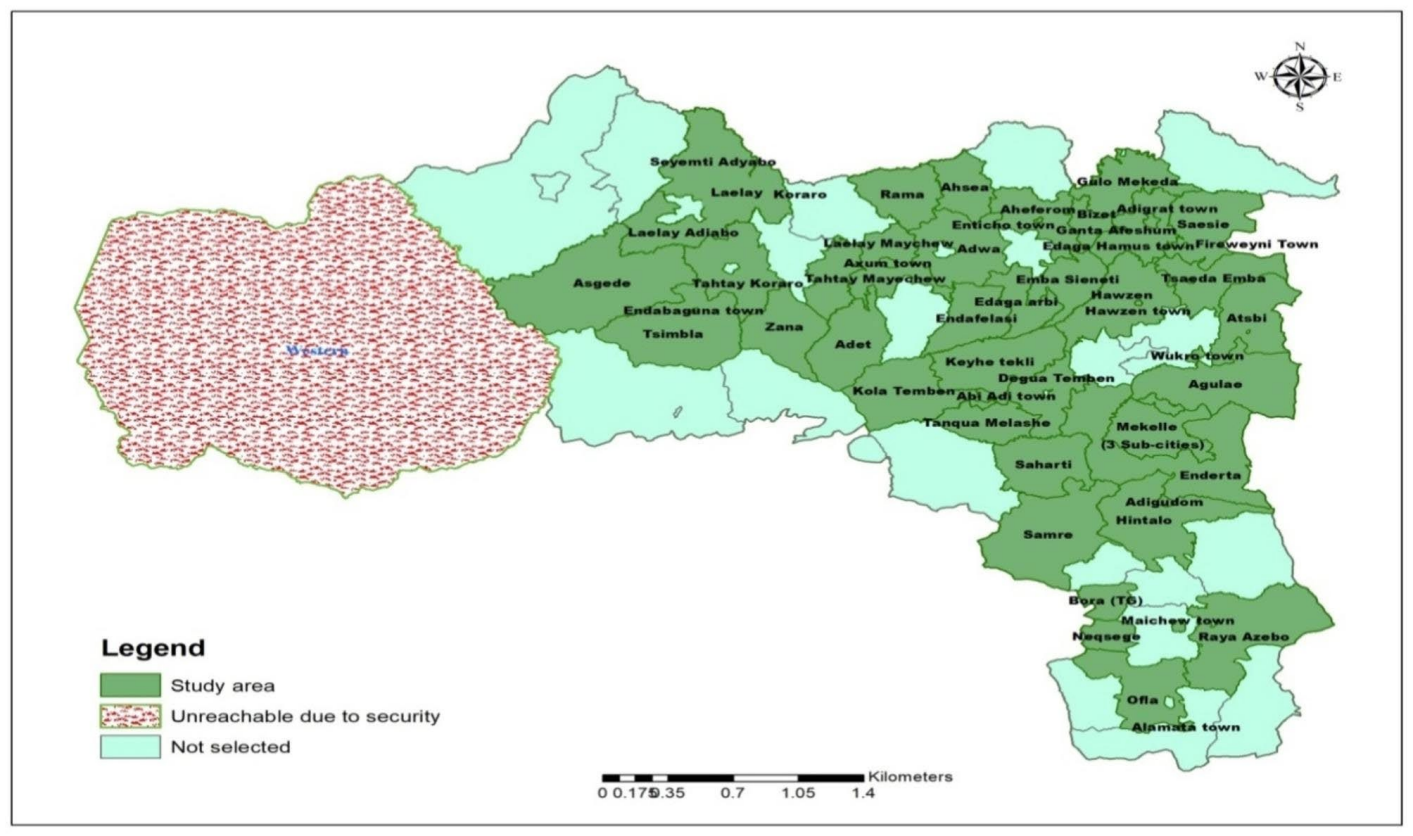
Map of study area

### Study design, population and period

This study is part of a sizable key performance health indicators (KPHI) regional survey conducted across all areas of Tigray with the exception of the western zone, where there is active conflict and was left out for security reasons. From 4 to 20 August 2021, a region wide community-based cross-sectional survey was carried out to gather information on a number of important performance indicators for child health. During the wartime period from November 2020 to June 2021, as part of the KPHI survey, caregivers of children under one year old were asked about whether their children had received the age-appropriate vaccinations, contracted one or more childhood illnesses and whether they had sought treatment for any of the stated childhood illnesses. The survey includes all caregivers of children under one years of age who have lived in the selected households for more than six months. After three attempts, households that were still closed were not included in the study, and the next household was taken throughout the data collection process.

### Sample size determination and sampling procedure

A total of 52 districts were randomly selected from 84 accessible districts in Tigray’s six administrative zones (excluding Western zone). Using an existing sampling frame in health posts prepared by health extension workers, the minimum calculated sample size was 4,160 from 52 districts where four *Tabias* were included from each recruited district and 20 households were recruited from each *Tabia*. Because additional households were needed other aims of the KPHI survey, the sample size is increased to 4,381 and we gather data from all recruited sample size.

#### Data collection tool, and quality control

The questionnaire was designed by reviewing the literature that is currently accessible on childhood illnesses, seeking treatment, and using child health services for illnesses and immunizations in conflict setting. To ensure quality, the questionnaire was translated first from English into Tigrigna and then back to English. Before the actual data collection, the caregivers were asked for their verbal consent. The tool was pre-tested, and any adjustments were made that were required. The study’s goal, data gathering strategy, confidentiality, and ethical concerns were all covered in the training provided to the data collectors. Supervisors were in charge of verifying, and evaluating the quality of the data as well as validating the questionnaire for accuracy and consistency.

### Indicators of child health service and measurements

Utilization of services, such as receiving routine vaccinations and seeking treatment for common childhood illnesses, was measured using the indicators for child health services. Caregivers were asked about whether their children had received the age-appropriate vaccinations throughout the eight-month conflict period from November 2020 to June 2021. Additionally, caregivers were questioned about if their children had ever experienced diarrhea, a fever, or a cough during the eight-month war and sought treatment for any of these illnesses. Age appropriate vaccine is a vaccine given in the first year of life which is a recommended time to administer standard childhood vaccinations in Ethiopia [11]. Seeking treatment is considered when children received care from medical professionals in their nearby healthcare facilities, such as health posts, health centers, and hospitals. The considerations to seek treatment includes cough accompanied with difficulty of breathing for acute respiratory infections, three or more loose or watery stools per day for diarrheal diseases and fever for febrile illnesses. A child is fully immunized when received one dose of BCG vaccine, three doses of polio vaccine, three doses of pentavalent vaccine, and one dose of measles vaccine.

### Data analysis

Data were first entered into the Epi-Data version 3.1 Software before being moved to the SPSS version 25 software for analysis [12]. Data were cleaned after coding, categorization, and variable merging. The study’s participants and critical variables were described using descriptive statistical analysis techniques like frequencies, percentages, and cross-tabulations, and they were then displayed as tables and figures. To determine whether there was a difference between the participant groups, a Chi-square test was utilized.

### Ethical considerations

Ethical clearance was obtained from the Institutional Review Board (IRB)of College of Health Science at Mekelle University, Tigray (Ref: MU-IRB 1906/2021). Each participant was informed about the purpose of the study prior to the interviews. We advised study participants about the voluntary nature of their participation and that they had the right to withdraw their participation at any time, without any consequence. We assured them the data or information that they provided during the interview was confidential and unidentifiable.

## Results

### Socio-demographic characteristics

We attained 100% response rate partly because we visited closed households three times replaced the immediate next household if still closed. The high demand for health service was a facilitator for our data collection. A total of 4,381 caregivers of children under one were included in the study. Of these, about 41% (1,813) of the children were 0–5 months old while the rest were 6–11 months old. Approximately one third (1,484/4,381, 33.9%) of children under 1 years old resided in an urban setting. Male children under 1 years old age account for 48.1% and nearly 75% of the caregivers were females.

### Vaccination upatake during the warperiod

A total of 61.3% (2,687/4381) of the children under the age of one received at least one vaccine, compared to 39% (1,694/4381) of the infants who received none of the basic vaccines. In addition, 20% (271/1353) of the children under one years of age received all recommended vaccinations (Table 1).

**Table 1:** Uptake of childhood vaccines among children under 1 year old during the Tigray war from November 2020 to June 2021

Vaccines received	Numbers of eligible Children	Number of children received vaccination	Percent of children received vaccination
Received no vaccine	4381	1694	38.7
BCG	4381	2427	55.4
OPV 0	4381	1183	27.0
OPV 1	4116	2393	58.1
OPV 2	4073	1802	44.2
OPV 3	3735	1315	35.2
Penta1	4116	2383	57.9
Penta2	4073	1796	44.1
Penta 3	3735	1316	35.2
PCV1	4116	2272	55.2
PCV2	4073	1747	42.9
PCV3	3735	1266	33.9
Rota1	4116	2332	56.7
Rota 2	4073	1751	43.0
Measles	1353	485	35.8
Fully vaccinated	1353	271	20.0

BCG = Bacillus-Calmette-Guerin, OPV = Oral Polio Vaccine; PCV = Pneumococcal Conjugate Vaccine

**Fig. 2:**
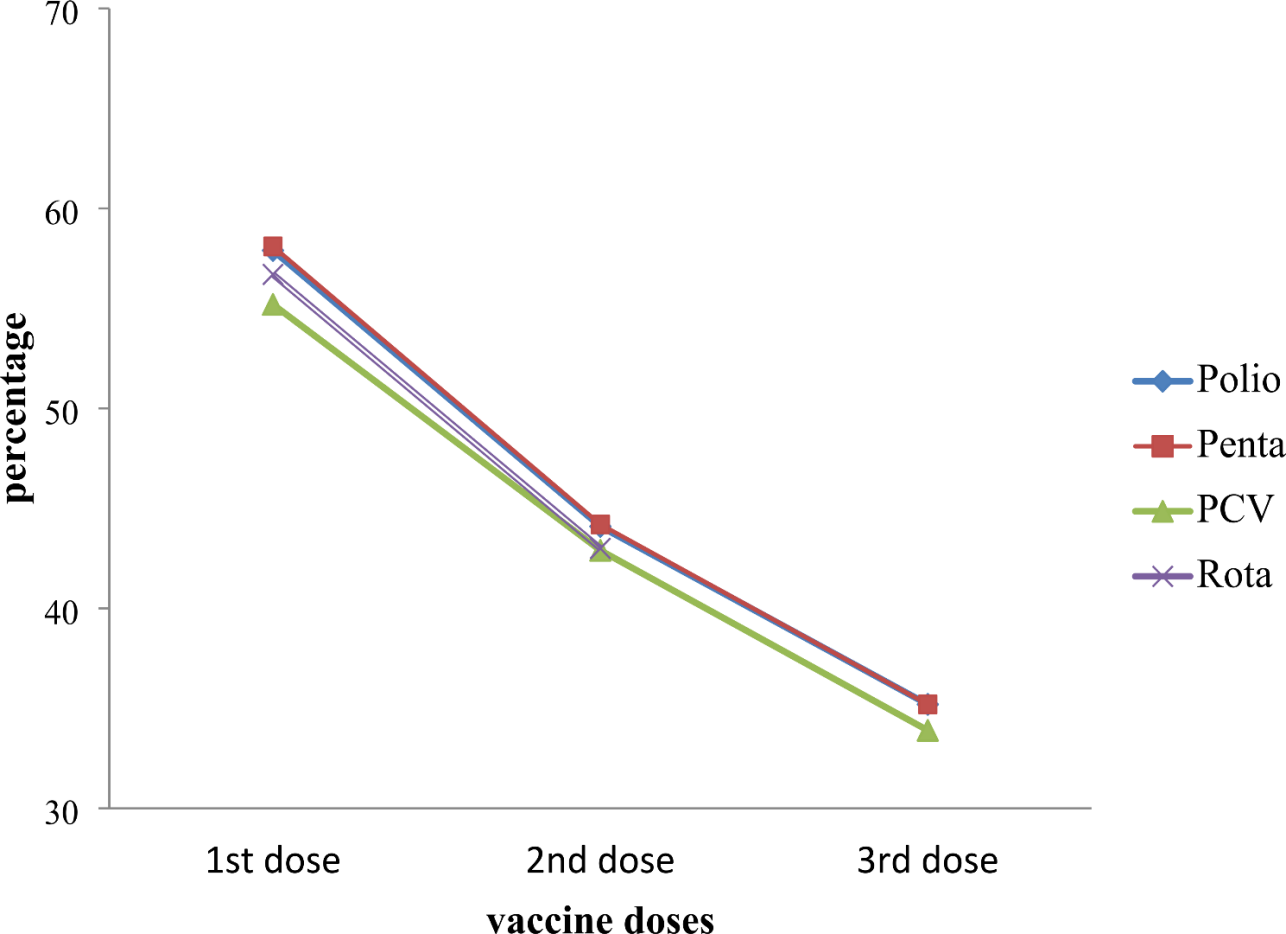
The uptake of subsequent childhood vaccine doses during Tigray war from November 2020 to June 2021. PCV = Pneumococcal Conjugate Vaccine

As shown in Fig. 2, the first doses of Penta (Diphtheria, Pertussis, Tetanus, Hepatitis B, and Haemophilus Influenza Type B vaccine), OPV (Oral Polio vaccine), PCV (Pneumococcal Conjugate Vaccine), and Rota (Rotavirus) were given to a range of 55.2–58.1% of infants. Children under the age of one received the second dose of the Penta and OPV vaccines in 44.1% and 44.2% of cases, respectively, while 42.9% and 43% of children received the PCV and Rota vaccines. According to Fig. 1, only 35.2% of infants under the age of one received the third Penta and OPV dose. This demonstrates a 23% decrease in the number of Penta and OPV vaccine recipients who received their third dose compared to their first. The third PCV dose’s absorption fell by roughly 21.3% when compared to the second dose.

Children under the age of one who resided in urban areas were more likely to receive vaccinations than their peers in rural areas. At a p-value of 0.001, the proportion of completely immunized children was 747(69%) in urban areas versus 335(31%) in their rural counterparts. As indicated in Table 2, urban children 312(64.3%) received the measles vaccine at a higher rate than their rural counterparts 173(35.7%).

**Table 2:** The uptake of childhood vaccines by residence during the Tigray war from November2020 to June 2021

Vaccine uptake	Residence		Chi-square test (P-value)
	Rural (%)	Urban (%)	
BCG (n = 4381)			< 0.001*
Yes	1221(50.3)	1206(49.7)	
No	1676(85.8)	278(14.2)	
Rota2 (n = 4073)			< 0.001*
Yes	792(45.2)	959(54.8)	
No	1874(80.7)	448(19.3)	
OPV3 (n = 3735)			< 0.001*
Yes	509(38.7)	806(61.3)	
No	1930(79.8)	490(20.2)	
Penta3 (n = 3735)			< 0.001*
Yes	512(38.9)	804(61.1)	
No	1927(79.7)	492(20.3)	
PCV3 (n = 3735)			< 0.001*
Yes	500(39.5)	766(60.5)	
No	1939(78.5)	530(21.5)	
Measles (n = 1353)			**<** 0.001*
Yes	173(35.7)	312(64.3)	
No	659(75.9)	208(24.1)	
Fully vaccinated (n = 1353)			< 0.001*
Yes	85(31.4)	186(68.6)	
No	747(69.0)	335(31.0)	

* Statistically significant at P value 0.001 BCG = Bacille Calmette-Guerin OPV = Oral Polio Virus; PCV = Pneumococcal Conjugate Vaccine

### Magnitude of common childhood illness and treatment seeking behavior

During the war, 4,301 children under the age of one reported experiencing one or more childhood diseases. After defining multiple responses, 50.1% (n = 2,154) of the children in this age group developed at least one illness. Cough was the most common illness recorded (1817/2154; 84.3%), followed by fever (1398/2154; 64.9%), and diarrhea (1113/2154; 51.7%).

Rural children had a greater prevalence of illnesses like diarrhea, fever, and cough, which affected 878 (78.1%), 1003 (71.7%), and 1218 (67.0%) of them, respectively. The mothers who did not visit PNC during the conflict were also more likely to have these reported childhood illnesses, which account for 875(78.6%) and 1081(77.3%) of diarrhea and fever, respectively. As shown in Table 3, residence and PNC attendance had a substantial impact on getting diarrhea and fever (P-value 0.001).

**Table 3:** The cross tabulation of childhood illness and treatment seeking by area of residence and PNC attendance during the Tigray war from November 2020 to June 2021

Variable	Residence		(P-value)	PNC attendance		P-value
	Rural (%)	Urban (%)		Yes	No	
**Childhood illnesses**						
Diarrhea (n = 4381)						
Yes	878(78.9)	235(21.1)	< 0.001*	238(21.4)	875(78.6)	< 0.001*
No	2019(61.8)	1249(38.2)		995(30.4)	2273(69.6)	
Fever (n = 4381)						
Yes	1003(71.7)	395(28.3)	< 0.001*	317(22.7)	1081(77.3)	< 0.001*
No	1894(63.5)	1089(36.5)		916(30.7)	2067(69.3	
Cough (n = 4381)				(n = 4,128)		
Yes	1218(67.0)	599(33.0)	0.150	489(26.9)	1328(73.1)	0.068
No	1679(65.5)	885(34.5)		744(29.0)	1820(71.0)	
**Seeking Treatment**						
Diarrhea (n = 1,113)						
No	649(73.9)	97(41.3)	< 0.001*	116(48.7)	630(72.0)	< 0.001*
Yes	229(26.1)	138(58.7)		122 (51.3)	245(28.0)	
Fever (n = 1,398)						
No	745(74.4)	147(37.1)	< 0.001*	142(44.8)	750(69.4)	< 0.001*
Yes	257(25.6)	249(62.9)		175(55.2)	331(30.6)	
Cough (n = 1,817)						
No	749(61.5)	408(68.1)	0.06	326(66.7)	831(62.6)	0.111
Yes	469(38.5)	191(31.9)		163(33.3)	497(37.4)	

* Statistically significant at P value 0.001PNC = Postnatal care

In comparison to rural, urban caregivers sought treatment more frequently for fever 249(62.9%) and diarrhea 138(58.7%). Only 257 (25.6%) and229 (26.1%) and of the caregivers who live in rural areas sought treatment for fever and diarrhea, respectively. During the war, mothers who attended PNC were more likely to seek medical attention for fever (55.2%) and diarrhea (51.3%). At a p-value of 0.001, residence and PNC attendance had significantly influenced the decision to seek treatment for diarrhea and fever.

## Discussion

This study assessed the prevalence of childhood illnesses and the availability of child health services in Tigray between November 2020 and June 2021. Only one in five children in this study had all the necessary vaccinations for their age, and 39% of children received no vaccinations at all, according to the vaccine status of this study. The first dose of Penta, Polio, and PCV was administered to more than half of the study’s young participants. Despite the fact that information on vaccination coverage was provided for children aged 12-to-23 months in the EDHS 2019 [13], the percentage difference between children received all basic vaccine at any time (44%) and those who received all vaccines in the first year of life (40%) is relatively small. Children received all basic vaccinations in Tigray before the war were three times higher than the finding in this study [13]. Moreover, percentage of vaccine uptake was more than two times higher before the war compared to the war period [14]. These suggest that the war had a substantial impact in disrupting the uptake of child immunization services.

The rapid decreases in vaccine uptake are related to war [14, 15]. Prewar vaccination uptake was roughly twice as high as postwar vaccination uptake for BCG, the third dose of Penta, OPV, and PCV. The first eight months of the war in Tigray caused substantial health system damage, which disrupted the provision of child health services [13].

The destruction of healthcare infrastructures, theft or vandalism of medical equipment, forced relocation of the health workforce, and attacks on the workforce are all contributing factors to the dramatic drop in the uptake of child health services [16, 17]. Other war-torn nations reported partial or complete disruptions in immunization services [18–22]which makes children more susceptible to diseases that can be prevented by vaccination. For instance, the current level of OPV3 vaccination coverage is comparable to the level 22 years ago, when there were many documented instances of polio. This suggests that because of the war and siege, many children in Tigray are at a greater risk of contracting diseases that can be prevented by vaccination [23].

In this study, vaccination coverage in urban areas remained more than twice as high as in rural areas. In particular, the health posts and health centers are used by rural communities for immunization of children. The war’s attacks on these primary healthcare facilities severely disrupted the provision of child health services [24].Consequently, many kids continued to be unvaccinated against diseases that could have been prevented and resulted in death or severe disability [25].Furthermore, children from Tigray’s rural and urban areas may experience long-lasting and permanent effects on their health and education as a result of this exposure.

Cough was the main contributor to the most typical childhood diseases, followed by fever. More than two third of cases of these common pediatric ailments were disproportionately prevalent among rural people. Compared to children in metropolitan areas, children in rural areas experience a higher burden of sickness but seek less care or guidance for it. Globally, common childhood infections like pneumonia, fever, and diarrhea continue to be major causes of child death. The impact of these illnesses is exacerbated by war, which also stops all measures that improve child health services [26]. When compared to the prewar period analyzed among children under the age of five, the proportion of infants under the age of one who experienced diarrhea and fever in the current study was more than twice. Additionally, these children reported coughing six times more frequently than children did in the years prior to the conflict [27]. If children under the age of five were considered, the percentage of children with certain childhood illnesses could be larger than it is in the current data.

Children are more likely to die from diarrhea and other sanitization-related illnesses than from the war itself [28]. While it can be difficult to access clean water sources during a conflict, occasionally armed groups purposefully attack water and sanitation facilities as weapons of war. Supplies needed for sanitation or to filter water are also made difficult by war. The rural community accounted for the majority of the childhood diseases reported in this study. The lack of sanitation facilities, safe and sufficient water, and restricted mobility to receive preventive services during the conflict may have been contributing contributors to this difference. Due to the fighting, 54% of the water points in Tigray were damaged, denying access to 3.5 million people [29].In fact, compared to urban areas, fighting was more intense in the first eight months of Tigray war in rural.

Mothers of children who did not receive postnatal care also had childhood illnesses three times more frequently than those who did. This underlines the fact that postnatal care is a time when a woman receives knowledge on all aspects of childcare, such as vaccinations, diet, hygiene, breastfeeding, and getting medical treatment as soon as possible when unwell [30]. Most women skip the education and counseling services in locations with very low postnatal coverage, which increases the burden of childhood illness [31]. In accordance with this, mothers who sought treatment when their children had diarrhea, fever, or a cough were roughly twice as likely to have attended postnatal care. This may be related to mothers’ inspiration to take care of their children due to the counseling and information provided during postnatal care visits. In Tigray, limited access and availability of maternal health services during the war caused, postnatal care to decline from 73%in the prewar to 19% during war [16].

The study was carried out right after the first eight months of the war and covered a wide geographic area with a sizable sample size. However, due to the fact that these territories were controlled by the allied forces (Ethiopian Defense Forces, Amhara Regional Government Forces, and Eritrean Defense Forces), a sizable number of districts in the Western zone of Tigray and some Tabias in the Eastern zone were left out of the research. Because of this, it is anticipated that child healthcare services will be severely compromised. It is possible that the level of harm to the health system under occupation will be greater than the damage that has already occurred in other areas of Tigray. Ignoring these areas might lead to underestimate the coverage of child health services during the Tigray war. Although mothers made up the majority of the study’s respondents, any household member 18 years of age or older was also eligible to participate, which raised the possibility of bias since these respondents might not have the same knowledge of childhood illnesses or treatment-seeking experiences as mothers. The recall period in the current study was eight months (during the war period from November 2020 to June 2021) which might lead to recall bias.

## Conclusions

The Tigray war, which is worse in rural areas, has significantly reduced the uptake of childhood immunizations. The prevalence of common childhood illnesses was intolerably high, disproportionately affecting children in rural areas. These childhood illnesses also affected children more frequently when their mothers did not receive postnatal care. Caregivers from urban residents and mothers who attended PNC sought treatment for children’s illness more often than their counterparts did. This study suggests that communities affected by war build basic child health services. Additionally, a dedicated effort is required to look into the underlying factors that contribute to childhood illnesses associated with war as well as ways to receive basic care during war.

### Electronic supplementary material

Below is the link to the electronic supplementary material.

Supplementary Material 1

## Data Availability

The data on which the conclusions of the manuscript rely are available in the paper. All datasets cannot be shared publicly because of sensitive information regarding the interviewees (mothers in conflict setting) and the restriction set by the research ethics committees. Data presented in this study are available on request from the corresponding author for researchers who meet the criteria for access to confidential data.

## List of abbreviations

BCG: Bacillus Calmette–Guerin
KPHI: Key performance health indicators
OPV: Oral Polio Virus
PCV: Pneumococcal Conjugate Vaccine
PNC: Postnatal care
WHO: World Health Organization
