# Supplementary material for: Child health services and armed conflict in Tigray, North Ethiopia: a community-based study
Source: Confl Health. 2023 Oct 5;17:47. doi: 10.1186/s13031-023-00545-6 (PMC10557173; doi:10.1186/s13031-023-00545-6)
Supplement: Supplementary file 1 — Supplementary Material 1 [file 13031_2023_545_MOESM1_ESM.pdf]

Rapid Assessment on the Key Performance Indicators of the Health Service Delivery System in Tigray

**Part – one : General information**		
**RN**	**Question**	**Response**
1.1	Zone	
1.2	Woreda	
1.3	Tabia	
1.4	Village	
1.5	Respondent sex	1=Female<br>2=Male
1.6	Name and signature of data collector	
1.7	Name and signature of supervisor	
1.8	Date of survey	|___|___| / |___|___| / |___|___| dd / mm / yy

**Part – two : Maternal Health Services (For Pregnant and Mothers with under one children)**

**Part – Three: Child Health Services (for under one children only)**			
**Age of the child in months:** --------------------<br>**Sex of the child:** Male/Female			
**Immunization**<br>(Record all the immunization status and date for each vaccine from the immunization card)			
**RN**	**Did the child receive the following Vaccine/s?**	**No = 0<br>Yes = 1**	**Date**
3.1	BCG		

3.2	POLIO 0		
3.3	POLIO 1		
3.4	POLIO 2		
3.5	POLIO 3		
3.6	PENTA 1		
3.7	PENTA 2		
3.8	PENTA 3		
3.9	ROTA 1		
3.10	ROTA 2		
3.11	PCV 1		
3.12	PCV 2		
3.13	PCV 3		
3.14	MEASLES		
Did (NAME) receive any additional vaccine via campaigns?			
3.17	Polio		
3.18	Measles		
3.19	Meningitis		
3.20	Hepatitis		
3.21	Others (specify): ___________________		
**Did your child contract one or more of the following childhood illnesses?**			
3.22	Has any child under one years of age had **diarrhoea** in the last 8 months?	1=No<br>2=Yes	
3.23	Did you seek advice or treatment for the **diarrhoea** from the nearby health facility?	1=No<br>2=Yes	
3.24	Has any child under one	1=No	

	years of age had **<u>fever</u>** in the last 8 months?	2=Yes	
3.25	Did you seek advice or treatment for the **<u>fever</u>** from the nearby health facility?	1=No<br>2=Yes	
3.26	Has any child under one years of age had **<u>cough</u>** in the last 8 months?	1=No<br>2=Yes	
3.27	Did you seek advice or treatment for the **<u>cough</u>** from the nearby health facility?	1=No<br>2=Yes	

NB: The PNC attendance is taken from part two (Maternal health service)
